# Collectanea: Pathology and Practice

**Published:** 1835-07-01

**Authors:** 


### COLLECTANEA.

### PATHOLOGY AND PRACTICE.

### REPORT OF THE SURGICAL AND OPHTHALMIC INSTITUTION OF THE UNIVERSITY OF BERLIN, FOR THE YEAR 1833.

This instructive account is contained in the first part of the 22d vol. of *Gräfe and Walther's Journal.* It is called merely an extract from the report; but, as it extends to seventy-five pages, we must still farther abridge it for the benefit of our readers.

*Summary view.* The number of patients treated in the year 1833, partly at their own houses, and partly in the hospital, was 1524; of whom, 1046 were surgical, and 478 ophthalmic. The total number of operations was 455; the surgical ones being 375, and the important ophthalmic ones 80.

*Removal of the Margin of the Eyelids along the whole row of Eyelashes.* This was performed on both eyes of a young man, on account of dystichiasis, causing inflammation of the eyes, with pannous cloudiness of the cornea. It was performed quickly, with comparatively little pain, without any injury to the shape of the eyelids, and with a perfect attainment of the object aimed at.

*Formation of Artificial Pupil.* The operation for this purpose was performed five times. In two cases, the patients could not distinguish the light, and the operation was performed only at their earnest request. It was done because several cases have occurred, where, in spite of complete insensibility to light, vision has been restored. The two patients in question were not so fortunate, but the operation was completely successful in the other three. The instrument used was Gräfe's *coreoncion.*

*Extirpation of the Eyeball and both Eyelids.* In this case a neglected trichiasis, of long standing, had caused a fungous degeneration of the eyeball and eyelids, as far as the edge of the orbit, to such a degree, that the approach of cancer was at hand. The cure was completed within a month. The remains of the eyelids gradually approached one another, almost covering the orbit; and, although the lachrymal gland was left behind, there was not the slightest trace of the secretion of tears.

*Mr. Guthrie's Eye-ointment.* Von Gräfe speaks in the highest terms of this remedy. The original formula is ℞. Argenti nitr. gr. iv—x.; Ung. Cetacei, ℥i.; Liq. Plumbi Subacet. gtt. i—xv. M.

Our author uses it in a milder form, as follows: ℞. Argenti nitr. gr. iij.; Axungiæ, ʒij.; Liq. Plumbi Subacet. gtt. v. M.

He prefers it to a strong solution of lunar caustic in water, and not only employs it in diseases of the eye, but introduces it into the urethra in cases of torpid and obstinate gonorrhœa, in which it does not cause any great pain.

*Restoration of the Nose from the Skin of the Arm.* The author expresses his gratification that the Italian rhinoplastic art, after having been forgotten for centuries, was first revived in his institution, has now been practised there for more than eighteen years, and has been copied at home and abroad. He was also the first to introduce the Indian operation on the continent, which has been oftener performed in proportion, in spite of the disfigurement caused by the scar on the forehead, a fact which is explained by its being much easier. Gräfe has read much of improvements upon his method of operating, but thinks that few of them deserve the name; he has always, however, been ready to recognise real merit, and has therefore thankfully profited by Benedict's judicious improvement of the bandage. In other points he has retained all the essential parts of his early method, merely adopting those simplifications which time has, of itself, a tendency to produce; and he would, therefore, have been in the wrong, had he considerably departed from his former manipulations, in the case which he is about to narrate.

The patient was a girl, aged twenty-one, who had lost the whole of her nose from syphilis, with the exception of a small portion of the root. The skin of the forehead being unfavourable for the operation, and the skin of the arm strong, the Italian method was preferred. As the patient did not suffer any inconvenience from the position of the arm, the separation of the transplanted portion was delayed till the eleventh day. The formation of the septum narium took place in the fifth week; and, in the course of the third month the patient returned home with a very good nose. Erysipelas of the face occurred, which in this, as in some previous and similar cases, was actively and successfully combated by antiphlogistic treatment.

The paleness of the new nose was succeeded by the natural carnation of the complexion in the third month, which was unusually soon.

*Polypus of the Mouth, of unusual size, successfully treated.* A patient, who had long suffered from dyspnœa and dysphagia, was found to have a polypus in the left nostril; and another, apparently independent of the former one, which filled up the cavity of the fauces. The latter polypus had pushed forward the velum palati, and, occupying its whole breadth, projected for half an inch under its edge, and rose so high upwards, that its boundaries could not be ascertained. The posterior surface of the polypus lay upon the mucous membrane in such a manner, that, at its accessible edges, it was impossible to get between it and the floor on which it had formed for more than a quarter of an inch. From the union of these circumstances, the operation, though absolutely necessary, appeared uncommonly difficult. Von Gräfe, on considering the obstacles, determined to overcome them in the following manner: He first removed the nasal polypus (which was about as large as a plum,) with the forceps in the ordinary manner, in order to gain space for approaching the other one. As soon as the patient had recovered from the operation, and all traces of inflammatory reaction had disappeared, the operation was commenced in the following way. A strong doubled thread, an ell long, was passed, by means of a large bent needle, through the part of the polypus which projected from under the velum palati; the ends hanging out of the mouth were tied together in a knot, and then twisted round an ear, to keep them fixed. This was done partly in order to be able to extend the polypus forwards during the operation, and partly to be able to extract it quickly when its separation should be effected, and thus avoid all chance of suffocation from this heavy mass falling upon the glottis; an accident which happened to a patient of Professor Meckel. A silken cord, an ell and a half long, (such as Von Gräfe uses for ligatures,) was now passed round the head of Bellocque's tube, and the instrument was introduced as far as possible through the left nasal meatus. When it seemed to be in opposition with the anterior wall of the polypus, we pushed its spring between the velum palati and the polypus, until the head was visible, and, catching hold of the silk thread by means of the long-armed toothed forceps (which is employed for making the incision in the velum palati in the operation of staphyloraphy,) drew it out of the cavity of the mouth. This part of the thread being held fast by an assistant, Bellocque's tube (after withdrawing the spring) was taken out the same way that it had been inserted. The two ends of the thread now hung out of the nasal meatus. It was easy to foresee that, under existing circumstances, the loop would not be passed round the polypus at the first attempt; and a long thread was therefore fastened to the middle part of the ligature which was hanging out, so that, in case of the loop slipping over the polypus, it might be brought out again without the trouble of again introducing Bellocque's tube. After these preparations, the loop was widened within the cavity of the mouth by means of two small rods (*Führungsstäbchen*). It failed, however, several times in encompassing the polypus, because the loop could not be sufficiently widened. Von Gräfe succeeded at last by introducing both forefingers, and thus enlarging the loop. It was necessary to tighten the ligature two or three times a day. The polypus soon swelled so much as to impede respiration, and require incisions to lessen its bulk. This was still more necessary when sphacelus began, in order to further, as much as possible, the expulsion of the ichor. Meanwhile the cavity of the mouth was frequently rinsed with solution of chlorine, on account of the fetid smell. The polypus came away in three days, and, reduced as it was by suppuration, was three inches long, one inch eleven lines broad, and seven inches and a half in circumference. No medicine was given the patient afterwards, excepting an emetic, to relieve some gastric symptoms, probably caused by swallowing the sanies. His recovery was perfect.

*Spontaneous Separation of the Right Half of the Lower Jaw, with its Articulating Process.* The author begins by asserting, that necrosis of the alveolar processes is very common after scarlet-fever, when mercury has been given so as to produce salivation, especially in children. The mere abuse of mercury, without the scarlatina, rarely causes this disease. The remedy to be administered is fuming nitric acid, as in the following case. A girl, aged ten, had had scarlet fever and been salivated three years before, and had, in consequence, been attacked with constant pain in the jaw, and very fetid ulcers of the mouth. She had lost all the teeth of the right half of the lower jaw, and in the soft parts there were unclean and putrid ulcers, and the upper surface of the alveolar processes was laid bare to the same extent, so that the metal sound came against nothing but firm and yellow dead bone. The patient was ordered to take a drachm of nitric acid daily, in a pint of decoction of malt; she was also put upon the use of malt baths, and rinsed her mouth with a weak solution of chloride of lime, alternately with tincture of myrrh in water. The pieces of necrosed bone were gradually and cautiously removed, until one half of the body of the lower jaw was entirely removed, together with the alveolar processes, without any fresh necrosis arising. After the lapse of several months, while the medical treatment was still being continued with short interruptions, the posterior part of the jaw came away of itself, with the articulating head, which was unaltered in shape. While the pieces of bone came away, a cartilaginous mass, which gradually ossified, arose from the soft parts, and restored the power of moving and using the jaw.

This case resembles a very interesting one in the Memoirs of the Surgical Academy, in which both halves of the lower jaw, together with their articulations, came away by necrosis in an adult. It remains for time to show, says the author, whether there will be a fresh growth of teeth in the new jaw, as he has occasionally seen in similar cases, in which, however, the loss had been smaller.

*Extirpation of Bronchocele.* This operation was performed on two patients. The first was a young man, of two and twenty, in whom the tumour was not larger than a goose-egg, yet the dyspnœa and dysphagia were intolerable. Only eight arteries were tied during the extirpation, and the parenchymatous bleeding from the thin paries of tumour left behind was easily stopped by cold water. The patient left the hospital in six weeks, perfectly cured. The second patient was a delicate and weakly girl, aged twenty-five, who had suffered since childhood from a bronchocele, consisting of three distinct tumours. It was thought prudent to operate on the middle one only, which was accordingly done, with the utmost success; for not only was the patient dismissed in six weeks, breathing freely, but the lateral tumours diminished in size from week to week.

*Extirpation of a Steatomatous Tumour on the Abdominal Parietes.* It is unfortunate, says Von Gräfe, that divisions of the soft parts by operation have often been considered only in their mechanical relations, without a proper appreciation of their dynamic influence. The following case warns us of the danger of laying large surfaces bare in the vicinity of the great cavities, and much more of the risk of opening the thorax or abdomen; operations which have been undertaken of late years, with unjustifiable thoughtlessness, and without any reasonable motive. A fatty tumour, as big as a man's fist, lying under the obliquus externus, and continually increasing in size, caused the patient numerous inconveniences. The operation was performed with ease, but was followed by an attack of peritonitis. This was subdued, with difficulty, by three large bleedings, and the most powerful internal remedies; the greater part of the wound healed by the first intention, and the patient went out cured.

*Amputation of the Penis.* The patient was a man of sixty, who had been treated homœopathically for eight months, but in vain. The glans was affected with fungus hæmatodes, (*Schwammkrebs,*) and there was scirrhus extending nearly to the arch of the pubes. The operation was performed by ligature, and the patient left the hospital in four weeks, perfectly cured; nor was there any return of the disease, when he was seen several months afterwards.

*Lithotomy.* The lateral operation was performed in a man, aged fifty-eight. Von Gräfe's gorget was employed; the stone was of the size of a hen's egg; the operation was completed within three minutes. There was no hemorrhage of any consequence during the operation, but a considerable secondary hemorrhage took place two hours afterwards: the patient, however, improved from day to day, and was cured in a few weeks.

*Ligature of the Brachial Artery in the Elbow-joint.* The brachial artery of a robust man had been wounded in venesection. Plugging was found to be insufficient, and the parts grew too sensitive to bear the tourniquet. The patient came under the author's care three days after the accident. The arm was almost incapable of voluntary motion, considerably swelled from the fingers to the shoulder, painful on pressure, very tense, and lividly red with spots of blue. The temperature was everywhere higher than natural Not the smallest trace could be discovered of the pulse either of the brachial or axillary artery; and that of the radial could be felt but obscurely. Gräfe observes that, in a case of this kind, it would have been unadvisable to tie the artery high up, near the inner edge of the biceps; for, though the artery is easy to find there, this can be predicated of it only when the parts are in their normal condition. He therefore resolved to tie it in the elbow-joint, as he should there be guided by the jet of blood from the wounded artery. The ligatures came away on the seventh day. The pulsation returned above the ligature in the same proportion as the inflammatory swelling decreased. Not a trace was to be felt of the pulse at the wrist. The wound closed in the fourth week, with the return of sensation and the natural temperature. Motion, strength, and the power of moving the arm, were recovered in four months.

*Aneurism by Anastomosis.* The patient was a countryman, aged thirty, who had laboured since his second year under a swelling of the left hand, which had increased more and more. Strong pulsations were perceptible on different parts of the hand, and several pulsating, irregular, elastic cords were to be seen along the whole fore-arm, and in a less degree on the back part. The brachial artery was tied at the inner edge of the biceps, and a perfect cure was effected.

*Paraguay Roux.* Von Gräfe, during his last visit to France, found a remedy of this name, which was much extolled against the toothach. On his return he tried it himself, and found it excellent, though not infallible. It seems to have a specific influence over the alveolar nerves, and may therefore be useful in other affections as well as toothach. When applied to healthy teeth, the Paraguay Roux excites a sensation of gentle, not unpleasant warmth in the crowns, which seems to penetrate deeply and extend as far as the end of the root; while the particles which happened to touch the lips or tongue caused a pungent yet cool sensation, with increased flow of saliva, which rarely lasted more than five minutes. When the teeth were painful, the Paraguay Roux caused the same phenomena, and, with very few exceptions, alleviated the pain; the duration of this relief, however, was very different; sometimes it lasted five or ten minutes, and sometimes several hours. When the pain returns, the application is repeated, and commonly succeeds in finally removing it, the third, sixth, or eighth time of using, without injuring the teeth, gums, or mucous membrane in the smallest degree, or leaving any unpleasant traces behind. Even when it was not a case of idiopathic neuralgia of the teeth, but depended on inflammation, rheumatism, or decay of the root, and was not cured by the appropriate treatment, this remedy maintained its efficacy. When the tooth is hollow, a few drops of the medicine are to be poured upon a bit of German tinder (Boletus igniarius), with which the cavity is to be filled up.

When the surface of the teeth is not carious, or when the pain extends to a whole row, the undiluted remedy is to be put upon the crowns by means of a brush, or else from fifteen to twenty drops diluted with a tablespoonful of water, are to be held in the mouth for several minutes, upon the affected spot. When it is used internally as an antiscorbutic remedy, ten drops are to be taken in wine every morning and evening, and the dose is to be increased by three drops daily, until thirty-six are taken. Von Gräfe has not yet tried its antiscorbutic virtues himself.

The Paraguay Roux is a patent remedy prepared at Paris by Roux and Chais; but Béral affirms that it is merely an alcoholic solution prepared from the Spilanthes oleracea, and Gräfe thinks that there is no difference between the patent tincture and Béral's. The green colour of both probably depends on spinage juice. The Spilanthes Brasiliensis and the Spilanthes fusca seem to have the same properties. The S. oleracea is also known by the name of Para Cress.

*Carrageen Moss.* (Chondrus crispus, *Grev.* Fucus crispus *Linn.* Sphærococcus crispus *Agardh.*) The author became acquainted with this new remedy during a visit to England, a couple of years ago. He speaks of its demulcent and nourishing qualities in the highest terms, and affirms that it almost uniformly lessens the colic pains attending several affections of the intestinal canal. We cannot quite agree with him, when he says that the jelly made from it is free from any unpleasant taste, as it certainly has a slight fishy flavour. Perhaps, however, some of the additions which Gräfe recommends, such as bitter almonds, orange peel, orange flower syrup, &c. may be sufficient to overpower this. The following are the formulæ which he prefers.

℞. Carrag. elect.* et concis. ʒ ss.; Lactis recentis ℥ ix.; Coque ad remanent. colat. ℥v. adde; Sacchar. albiss. ℥ss.—j.; Aquæ amygd. amar. concentr. ℈j. M. et refriger. d.

℞. Carrag. elect. et concis. ℥iss. Coque c. aquæ fontan. ℥xij. ad remanent. colat. ℥v. adde: Syrup. rub. Idæi, ℥iss.—℥ij. M. refriger. dentur.

*Devis's* [*Davis's*?] *Solution of the Hydriodate of Potash.* Gräfe has used this remedy with success in arthritic, scrofulous, and syphilitic diseases, and has found it of great service, when sarsaparilla and mercury had been employed in vain. It never caused any affection of the chest, or any important attenuation of the patient, or weakening of the digestive organs. The prescription is as follows: ℞. Potassæ Hydriod., ʒss.—ʒj.; Iodines, gr. ss.—j.; Aquæ destill. ℥viij.; Syr. Papav. alb. ℥ss. M. d. s. A tablespoonful is to be taken three times a day, and the dose is to be gradually increased.

*Sarlandiere's Moxas.* These are made at Paris, by Sallé, an apothecary, probably from the lanugo of the Artemisia vulgaris.

* The fucus, as met with in commerce, is to be stirred in cold water for five minutes, and, being then dried, is to be called *Carrageen electum.*

They do not require constant blowing, like other moxas; they burn with less smoke, and in intensity they hold a medium between the mildest and most violent of those formerly used.

*Remarkable Cure of Epilepsy.* A servant girl, æt. twenty-four, had long suffered from hysteria, which, by the diminution of the catamenia, had been aggravated into epilepsy. Von Gräfe determined to endeavour to increase the catamenial flow by a mixture of aloes, rhubarb, and savine, and to act upon the imagination of the girl by inserting *nephritic* stones in the arms, and allowing the wounds to heal up and enclose them. A small serpentine stone, three or four Paris lines in diameter, was inserted into each humerus, and the wounds were healed by the first intention. It is remarkable that the first fit which occurred after this operation, and which was delayed much beyond the usual time, took place when one of the stones, which had not been inserted sufficiently deep, was just ready to fall out. It was taken out, and reinserted in another part of the arm. The catamenia became more abundant, and there was no return of hysteric or epileptic paroxysms.

*New Compress Instrument for deep-seated Hemorrhage, occurring in the Lateral Operation for the Stone.* We pass over in this place, says our author, the hemorrhages that occur from the division of vessels which can be reached by ligature, and mean to treat of those alone where, from the depth of the wound, a ligature cannot be applied. In these latter cases, it is rarely possible to decide with certainty whether the hemorrhage proceeds from a single large artery, or from numerous small vessels of the perineum, neck of the bladder, or bladder itself; for calculous disorders, by their continual irritation, often develope a vascular plexus not belonging to the normal anatomy of the part. In such hemorrhages, when repose, approximation of the thighs, the use of acidulous drinks, cold injections into the incision, and the application of ice to the perineum and region of the bladder, have all failed, we have no resource but the use of *tampons*. They are commonly applied by means of a female catheter, covered with German tinder, and pushed as far as the incision into the bladder. Richerand used the catheter, but without this covering, placed it along the lower angle of the incision, and endeavoured to fill up the space between it and the upper part of the incision by plugs of charpie pushed in to a considerable depth. As narrow tubes soon become obstructed by coagulated blood, Benjamin Bell proposed a hollow metal cylinder, somewhat flattened, and about an inch wide, which was covered with German tinder before using. All these apparatuses form cylindrical tampons, which are equal in diameter at top and bottom. If they are large enough to press the deep-seated vessels sufficiently, their introduction causes sensible pain, and the more external parts of the track of the incision suffer, without an object, the same degree of pressure which is required for the deeper points alone. Moreover, it must be considered that, from the equality of their superior and inferior diameters, these cylinders are very apt to slip out, unless remarkably well fastened, from the parietes of the incision giving way under the pressure; a thing which generally happens soon. To avoid these inconveniences, our author had an instrument made fifteen years ago: it is a tube constructed of several silver plates, put together so as to form a cone, which is capable of being broadened at its point. The small end of the instrument is introduced while shut, and, by opening at the spot required, presses upon the vessel. Gräfe gives several figures representing this ingenious contrivance; but, as we have not this advantage, we think it needless to perplex our readers by a further description of the instrument.

*Creosote.* Gräfe has not seen much benefit derived from the use of this preparation: it destroyed, indeed, the smell of ichorous and putrid ulceration, but not more readily than was effected by the application of simple tar-water, cinchona, pyroligneous acid, chloride of lime, or charcoal. In impetigo *(Flechten)*, and in scrofulous and phagedenic ulcers of various kinds, it was not remarkable even as a palliative; far less did it cure them. Pure creosote, applied to the cancer of the skin, produced changes of a doubtful kind, which never led to recovery; and this in cases where, after its fruitless application, the author soon effected a cure by means of his corrosive ointment. Nor did its internal use answer the expectations which had been cherished. In cases of chronic eruptions of long standing, and spreading impetigo, it was prescribed in doses of half a drop, gradually increased to fifteen drops twice a day, without any effect, good or bad. When dropped upon cotton, and inserted in the hollow of carious teeth, it sometimes removed the pain, but could not be relied upon. Gräfe has therefore, as yet, no confidence in the medicinal virtues of creosote.

*Graham's Remedy against Cancer.* This seems to consist chiefly of phosphate of iron. Many preparations of iron have long been employed, both internally and externally, against cancer, especially the carbonates; but Gräfe has never seen any essential advantage derived from their use, and Graham's composition is not better than the others.

### AN OPIUM-EATER.

Dr. Christison in the second edition of his work on poisons has added some valuable remarks upon opium-eating (p. 626-629.) A remarkable trial which occurred after the publication of the first edition drew his attention forcibly to this subject; he found, he says, that practitioners and toxicologists generally possess little or no precise information on the subject, but he hopes that what he has to state will induce other persons to contribute their knowledge towards filling up so important a blank in medico-legal toxicology. A wish expressed by so eminent a writer may perhaps be considered an excuse for my giving the following account of an opium-eater, though I must confess that it contains nothing very striking.

April 6th, 1835. W. B. a light porter, ætat. 51, was in the

Middlesex hospital about 24 years ago under Dr. Gower; his disease was acute rheumatism, for which opium was administered. He began opium-eating immediately on leaving the hospital, but the quantity he took was so small that it might be considered rather the continuance of his medicinal doses, than the commencement of opium-eating. At this time, half a drachm lasted about three weeks. This was gradually increased to a drachm and a half a week, or sometimes a little more, which has now been his dose for five years. The opium scarcely constipates his bowels; his urine is free, his appetite very good, his sleep sound, his pulse regular. He is not subject to headach, excepting a sick headach once a year or so. If he goes without his opium, he feels a sinking and gnawing in his inside. He says that he rarely takes spirits and but little beer. (I heard however from another and authentic quarter that he is far from abstinent with regard to spirits.)

In spite of the favourable account which he gives of himself, the appearance of W. B. is such as to hold out no encouragement to opium-eating. He is sallow, listless, and weak, and his tone is the very reverse of that of a man in good spirits. He says that his father, a medical man who died at the age of 66, had eaten opium in very large quantities for many years. Perhaps, then, part of his torpor may be hereditary. E. A. Domeier.

### CASES OF PARALYSIS OF INDIVIDUAL NERVES OF THE FACE. BY DR. CHRISTISON.

1. *Palsy of the Third Nerve, or Motor oculi; recovery under free venesection and mercurial salivation.* A stout blacksmith, of middle age, and much given to drinking, was admitted into the Fever hospital, supposed to labour under continued fever. During the confusion incident upon opening the hospital, and receiving a great number of patients before the appointment of a physician, the exact nature of his case was for some days overlooked. But a day or two after my appointment, my attention was called by Dr. W. Reid, then superintendent of the hospital, to a considerable squint of the left eye; upon which the following account was given by the patient.

Three weeks before his admission into the hospital, and immediately after a debauch, he first remarked that he saw double; and at the same time he was affected with ringing in the ears, headach and giddiness, which last symptom was particularly troublesome when he kept his left eye open. After a fortnight he had rigors, general soreness, and increase of headach; and for ten days after admission into the fever hospital, he had the symptoms of a mild attack of general fever, with continuance of double vision. Subsequently the two images gradually approached one another, till at length they coincided, and he saw single; and at the same time the general fever ceased. When first carefully examined a few days after that, it was observed, that the left eyeball was turned very much outwards; that he had no power whatever to turn it upwards, inwards, or downwards; that when desired to look up he merely rotated the eyeball on its horizontal axis inwards; and that the upper eyelid always hung over the ball, so as to be in contact with the lower, except when he was told to raise it, upon which he uncovered half only of the pupil. The pupils were equal in size and contractile, and when he closed either eye he saw quite well with the other. The vision with both eyes was not double. He had frequent diffuse headach, but no disorder in any other function.

For some days after this examination, the state of the affected eye varied, as he occasionally recovered imperfectly the movements of the eyeball for twenty-four hours at a time. But at length they were lost entirely. The treatment for ten days after the paralytic affection was remarked consisted in the occasional application of leeches to the temple, and of a blister issue behind the ears, together with frequent laxatives. No amendment, however, was procured from these measures.

Four or five days later, when he had been three weeks in the hospital, his headach began gradually to increase, and the pulse to rise; in a day or two more he complained of great general soreness of the head, and of shooting pains, numbness and diminution of muscular power in both limbs. Blood was therefore drawn from the arm to the amount of two pounds. It was very buffy, and gave immediate relief from the headach. Calomel was also prescribed in the dose of five grains four times a day, with a little opium, for the purpose of quickly affecting the mouth. When this had been continued four days, the affection of the limbs ceased, and the fever abated considerably; but a slight deviation was remarked in the tongue to the left, and in the mouth towards the right side, while at the same time the affected eye was turned more outwards than ever. On the sixth day of the mercurial treatment his gums became tender; and the calomel was therefore discontinued. A moderate salivation immediately followed. On the fourth day after the discontinuance of the calomel, there was an obvious improvement in the state of the eye, and in twenty-four hours more the improvement was considerable. The movements of elevation, depression, and adduction of the eyeball, could be performed to half the natural extent, and he could raise the eyelid so as to uncover two thirds of the cornea. At the same time the deviation of the tongue and mouth, the headach, and all his other complaints ceased.

From this time he improved rapidly and steadily, so that in a week after the lost movements recommenced they could be performed perfectly. The salivation lasted twelve days, and was always gentle. In twelve days more he was dismissed quite well, having been in the hospital seven weeks.

It is worthy of remark, that after the movements of the affected eye returned so completely, that this eye, when the other was covered, could allow a moving object briskly in any direction, some days more elapsed before the affected eye accompanied the other in its movements, when both were uncovered. For example, if the patient was directed to look at an object straight before him, he squinted the left eye outwards, and looked at it with the right only; but if he was told to look at it while the right was covered, he turned the left on it; and when the right was then uncovered, he directed both eyes on the object, and for a few seconds followed it with both, when it was moved before him.

I shall not attempt to explain the singular fact mentioned at the commencement of the case, as to the man first seeing double, and then remarking a gradual approximation of the two figures till they coalesced, and he saw single, while the squinting of the eye nevertheless continued. This is so different a phenomenon from what we should expect in the circumstances, that I shall not insist on the accuracy of the statement, because it was drawn up from the man's own account of what occurred some days before I saw him. Yet it is fair to add, that he always gave a very clear narrative of the progress of his illness, and never deviated in any particular from his first statements. In such a case, we should expect, not that the images would gradually approach and coalesce, but simply that the individual would gradually acquire the habit of abstracting his attention from one of the images, and so recover single vision.

2. *Palsy of the Portio Dura of the Seventh Nerve on both sides.* A young man of middle stature was admitted into the Fever-hospital on account of a smart attack of continued fever of the inflammatory type, but without any particular local inflammation. On the fourteenth day of the disease he got quickly better by critical sweating, the pulse falling from 120 to 72 in the course of a single night. Nothing occurred to interrupt convalescence till the end of the fourth week, when he complained of a sore mouth, for which a vinegar wash was ordered. In five days more, however, the man continuing still to complain of his mouth, a careful examination was made, when the dead stillness of his countenance at once attracted attention. The lips were completely palsied and could not be closely shut, the nostrils could not be curled, the upper eyelids could not be closed, and he could neither laugh nor whistle. At the same time the sensation of the affected parts was everywhere perfectly entire. He had not the slightest fever, no headach or local pain of any kind, except soreness of the mouth; and his only complaint, indeed, was of the dryness and soreness of his lips.

Low diet was ordered, blisters were applied behind the ears, and leeches immediately before them, and laxatives were frequently administered; but without the slightest advantage. About this time the last patient, with palsy of the motor oculi, recovered apparently under the operation of mercury. The same treatment was therefore applied in the present instance, and a smart salivation was brought on, which terminated in a copious impetiginous eruption over the whole face. The patient, however, did not derive the slightest benefit. All the parts supplied by the muscular portion of the seventh nerve on each side of the face continued in a state of perfect paralysis. After remaining three months in the hospital he was dismissed in the same condition; and I have never since been able to receive any information of the progress of his disease.

3. *Paralysis of the Portio Dura of the Seventh Nerve of the Right Side.*—When in the country last autumn, I was requested to visit a young man who was supposed to labour under palsy. His paralytic affection was of five or six days' standing; and it was preceded for some days by severe aching pain in the whole right side of the face. When I saw him the pain had ceased; but the whole right side of his face was completely paralyzed. In a state of repose it did not present any peculiar appearance, except considerable depression of the right eyebrow. But on careful examination, I found that he was unable to whistle, to curl up the right side of the nose, to elevate the right eyebrow, or to close the right eyelid, and when he laughed the mouth was drawn much to the left side. The right eye watered a good deal, and when told to close it, he closed the lids imperfectly, and turned the pupil upwards beneath the upper eyelid.

He complained of acute pain on pressure upon the cheek in the region of the exit of the infra-orbitary nerve, and likewise in the jaw at the exit of the inferior maxillary nerve upon the chin, also of *tinnitus aurium* when at work. He had never had headach, or any pain or swelling before or around the ear; neither was there ever any weakness of the limbs or arms, any material difficulty in speaking, or divergence of the tongue from the straight position when thrust out. The sensation of the cheek, too, was quite entire. The pulse was natural.

I recommended him to have a blister applied frequently before and under the right ear, and after following this plan of treatment to resort to a mild course of mercury, if he was not relieved. Six blisters were in consequence successively applied between the close of September and beginning of December, and during the latter part of that period mercury was given so as just to affect the mouth.

Some amendment was remarked after the application of the last blister, which extended from behind the ear to the chin, and acted very severely. Till then he experienced no improvement, and since then he has made but little progress. I am unable to state the precise amount of amelioration that has taken place; but I have been informed by the clergyman of his parish, that he can now close the right eyelids completely, but does so very slowly, that the other palsied actions of the muscles of the face appear to remain nearly as when I saw him, and that he has still acute pain on pressure on the cheek under the eye, or on the lower jaw near the chin, but that he continues entirely free of general palsy or any head symptom.

Aug. 18, 1834. The patient eventually got quite well.

4. *Palsy of the Optic Nerve?*—The next case to be mentioned

I have entitled palsy of the optic nerve; although I believe the propriety of the name is doubtful.

A middle-aged man, of meagre habit, and very deeply scarred with smallpox, was admitted in the summer of 1829 into the surgical department of the Infirmary, on account of chancres and bubo. The chancres soon healed; but the sore left in the groin after the suppuration and opening of the bubo was untractable. While in this state, he was attacked with dysentery, which at that time (autumn 1829) prevailed in the hospital, and was so fatal that about a fourth of those who were taken ill perished. Being transferred to the medical department on account of the dysenteric attack, he became my patient. At this time he had dysentery in its worst form, and although the acute stage was soon subdued, the symptoms nevertheless showed that extensive ulceration had taken place in the intestines; and he died six weeks after I saw him.

At first no attention was paid to the circumstance of his being blind of an eye, as the marks of severe confluent smallpox seemed to account for it sufficiently. But on his mentioning to me incidentally, that the sight was lost only two years before, careful inquiry was made; and it was then learned, that, on the occasion alluded to, he had been a patient in St. Bartholomew's Hospital, London, with severe headach, giddiness, feverishness, and incomplete palsy of one of his sides; and that he gradually got the better of these symptoms, but was at the same time attacked with inflammation of the left eye, which burst, and became totally blind.

It was natural in these circumstances to expect that some injury would be found in the course of the fifth nerve. This nerve, however, was found quite healthy so far as the sight and touch could determine. But the optic nerve of the affected side, between its exit through the orbit and its decussation with the opposite nerve, was not more than half the breadth of the other, and was gray in colour and flaccid in texture. Between the point of decussation and the thalamus of the opposite side it was of the natural whiteness, but softer and less than its fellow; and the thalamus itself was somewhat flattened. The brain was otherwise healthy, except that a very great watery effusion had taken place under the arachnoid coat over the whole external surface, which appeared to account for the severe headach and frequent incoherence remarked towards the end of his illness. There was no appearance of an old cyst or other disorder in the substance of the brain. The colon was covered to an enormous extent with ulcers in various stages of progress. The left eye was completely disorganized.

I am quite aware, that, in calling this case palsy of the optic nerve, I may err in taking for the cause of the destruction of the eye what was really its effects. Neither do I pretend to say, that there is much probability in the view here given. But it appears to me right to make known the particulars, because it is not impossible that diseases of other parts of the nervous system besides the fifth nerve may lead to destruction of the eye, and this is presumptively a case of the kind, the presumption being founded on the bursting of the eye having been immediately preceded by some organic disease of the brain, and being not associated as usual with disease of the fifth nerve.—*Edinburgh Med. and Surg. Journal.*

### ACCIDENTAL OCCLUSION OF THE VAGINA, FORMING AN OBSTACLE TO DELIVERY.

BY C. HOILLEMIN, D. M. P. OF AUX CAYES, HAYTI.

Madame — de —, when twenty-five years of age, had an exceedingly difficult labour, lasting three days; during which she had no other assistance than that of an inexperienced midwife. The external parts of generation, as well as the vagina, were attacked with violent inflammation, which was followed by an almost complete closure of the vagina, only a small opening remaining, scarcely sufficient to allow of the passage of a goose quill. She long suffered from incontinence of urine, and much difficulty in walking.

About June, 1830, Mad. —, then twenty-seven years of age, first consulted me. She was at that time suffering with nausea, loss of appetite, progressive increase of the abdomen, swelling of the breasts, &c. I immediately recognised all the symptoms of pregnancy at the third or fourth month, and informed Mad. — of it, who replied that it was impossible for her to be pregnant, since she could not cohabit with her husband, because her parts were closed, "*ses parties sont fermée,*" (this was her expression.) The husband, who was present, confirmed all that his wife had said. Nevertheless, I assured her, that she was undoubtedly pregnant, and I did my best to tranquillize her great uneasiness, for she incessantly repeated that it was impossible for her to give birth to her infant.

The 30th of December of the same year, Mad. — sent for me at midnight. Labour pains had just come on. On examination I found that the vagina was closed by a firm membrane, extending across it, and which was thickest laterally. Near the meatus urinarius, a kind of fleshy band originated, which was lost in the partition. In the centre of this last there was a round opening, scarcely large enough to admit a quill, and the margin of which was thick.

I proposed to Mad. — to divide the membrane closing the vagina, to which she consented. After the uterine contractions had continued for six hours, I took advantage of the moment when the membrane was pressed forward and downwards by the membranes and the head of the child, to divide the margin of the opening, and then inserting the index-finger of my left hand between the head of the infant and the partition; with my right hand, I passed the blade of a straight probe-pointed bistoury upon the finger which served as a conductor, and cut the membrane from within outwards, on the left side, to the extent of an inch, and then waited the effect of the renewal of the uterine contractions. After an hour, during which these were strong and frequent, the opening not enlarging, the membranous partition being constantly pressed down, I made another incision from within outwards on the right side, so that these two incisions formed a triangular flap, the base of which was towards the sacrum. The umbilical cord immediately protruded; the waters, which were discharged, were black, and exhaled a strong and disagreeable odour. Mad. — became covered with a cold sweat; had repeated faintings; her pulse was almost imperceptible, and the uterine contractions were infrequent. Suspecting that the child was dead, from there being no pulsation in the umbilical cord, and having great fears for the mother, I hastened to terminate the labour by delivering with the forceps. The child appeared lifeless; its surface was livid, indicating cerebral congestion. After dividing the cord, I allowed three or four ounces of blood to flow; I employed dry frictions over the cardiac regions, &c., and was not a little surprised to see the infant revive, as well as the mother, both of whom are at present in the enjoyment of perfect health.

Precautions were taken to preserve separate the parts which had been divided, and to prevent their reunion; the opening of the vagina was thus reestablished in its natural state. The triangular flap resulting from the two incisions gradually diminished, and at the end of two years no trace of it remained, and Mad. — could cohabit with her husband without experiencing any inconvenience.

This case appears to me to be interesting in a double point of view, both as respects the delivery and conception. I submit it to the profession, believing it to be not unworthy of their observation. —*American Journal of Med. Sciences.*

### DR. HOPE ON THE SOUNDS OF THE HEART.

Dr. Hope has lately appended to his work, on Diseases of the Heart, a Supplement on its Sounds; and, as the subject is one of great importance in the diagnosis of cardiac disease, we make no apology for reprinting the whole of the appendix.

"It has, I believe, been almost universally admitted, both in this country and abroad, that the experiments detailed at pages 21 and 30 fix the first sound of the heart on the contraction of the ventricles, and the second, on their dilatation. The experiments did not demonstrate the immediate causes of the sounds, and my inferential explanations of them (at page 48) were soon doubted. Some thought that the closure of the valves was the cause; and I readily cede the merit of originality in this supposition to M. Rouanet, Mr. Bryan, M. Bouillaud, Mr. Carlile, &c.: others, as Dr. Williams, ascribed the first sound to the muscular sound, on which I have (at page 47) given an opinion essentially substantiated by the subjoined experiments.* If my explanations were erro-

* The opinion of M. Magendie, who ascribes the sounds to the beat of the heart against the ribs and the spine, does not require grave notice, being refuted by the experiments, page 23 and 26; and by the subjoined experiments, No. 11: See also Hydropericardium, page 515.

neous, it was my duty to correct them; I therefore commenced in 1832 a new series of hospital researches on the living and dead subject, and soon satisfied myself that the first sound was loudest over the middle of the ventricles, and the second, over the sigmoid valves, and thence for a few inches upwards; also, that when a healthy subject was faint, the first sound lost its prolongation, and became short and smart like the second; whence I inferred that, in its natural state, it might have a compound cause, viz. the closure of the valves, and the motion of the blood, or the bruit musculaire.

The presumptions thus offered, that the valves were concerned in the production of the sounds, required corroboration by experimental and pathological evidence. Not having succeeded in satisfactorily imitating the second sound by injecting fluids retrograde into the aorta, I tried the expansion of membranes under water, and found that three inches of fine tape, two lines broad, held to the end of a stethoscope, and gently jerked under water, imitated the second sound, both the sounds in dilatation, and the double sound of the fœtal heart, to perfection. Hence it was more than probable that the sudden expansion of membranes so small as the sigmoid valves was sufficient to produce such a sound as the second.

It was not easy to meet with satisfactory pathological cases on this subject; as, to be conclusive, great disease of the valves on both sides of the heart simultaneously, seemed to be required. Case 18, page 580, was one of this kind; the mitral aperture being about a quarter of an inch, and the tricuspid half an inch in diameter; yet the second sound, though weak, was perfect and without a murmur. Now, had this sound been occasioned merely by the influx of the blood, or any other cause than the sigmoid valves, surely it would have been attended with a murmur.

R. S., Esq., whom I saw in consultation with Dr. Armstrong, had a prolonged bellows murmur over the sigmoid valves instead of the second sound. On examination, the orifice of the ossified and dilated aorta was found so much enlarged that the valves probably did not close it: hence a murmur from regurgitation, which extinguished the second sound. But why, it will be said, was the sound not produced by the pulmonic valves? True; therefore I did not consider the case conclusive. I have elaborate notes of three other similar cases; but as the patients are living the evidence is still less conclusive. I have, however, notes of the case of *Thomas Wood*, in the St. Mary-le-bone Infirmary, October 21, 1833, made by myself and by Mr. Hutchinson, late resident surgeon to the institution, separately, attesting that a murmur from regurgitation through the mitral valve *completely* drowned the first sound in the vicinity of the valve. Whence it might be inferred that a murmur in one set of sigmoid valves might possibly drown the natural sound of the other set.

On the whole, therefore, the presumptions were exceedingly strong in favour of the second sound being produced by the sigmoid valves; and the evidence on the first sound, more fully explained in the sequel, led me to establish the following presumptions; viz., that the first sound was *compound*, consisting, 1. Of the valvular flap; 2. Of an augmentation of this, either from *bruit musculaire*, or the motion of the fluid, or both* ; 3. Of the prolongation of the sound by *bruit musculaire*, or the motion of the blood.

These presumptions required to be proved. After much reflection, a mode of experimenting on the ass occurred to me, which, if practicable, would inevitably, I thought, prove conclusive; namely, after denuding the heart in the manner described at page 22, to work out the following propositions:

1. Is the second sound loudest over the sigmoids, and is it so *near* as to seem produced immediately under the stethoscope?

2. Is the first sound loudest over the two auricular valves respectively; and is it so near as to seem produced immediately under the stethoscope?

3. Place the origins of the aorta and pulmonary artery between the finger and thumb; apply the stethoscope on the heart near the sigmoids; instantly after the systole, close the arteries, so as to prevent the reflux of the blood and consequent expansion of the valves, and see whether this annihilates the second sound.

4. Relax the fingers during the interval of repose, and see whether this reproduces the second sound at its wrong interval.

5. Push the knuckle, or the auricle, into each auriculo-ventricular orifice, so as to prevent the expansion of the valves, and see whether this annihilates the first sound.

6. Introduce a bent needle into the aorta, and hold open one or more of the semilunar valves, so as to permit free regurgitation. Notice whether this occasions a murmur with the second sound. The pressure of the aortic system being thus thrown on the ventricle, will it close the mitral valves? See whether this annihilates the first sound on that side.

To pave the way for the experiments on the ass, I first, assisted by Mr. H. J. Johnson, made trial on a rabbit, poisoned with woorara. Though the heart acted vigorously for an hour, and we *could perfectly hear both sounds* by applying the small end of a thin stethoscope, the organ was too diminutive, and its movements too quick, to admit of our appreciating modifications of the sounds. I then proceeded to a trial on the ass, at Mr. Field's, Nov. 3, 1834, and, as on former occasions, put a copy of the above propositions into the hands of the friends invited (Drs. Davies and Williams, Mr. Johnson and Mr. Field). From an accident, the operation failed, the action of the heart being nearly suspended by the time the organ was exposed. The valves, indeed, were hooked back and the sounds heard, but with unsatisfactory results. I made

* It will presently be seen that another cause, the sound of muscular extension, was the principal source of this augment.

arrangements to renew the experiments immediately; but, from unforeseen and unavoidable causes, they were delayed till the ensuing February, when Dr. Williams and myself resumed them conjointly. Woorara was employed, and the heart, when denuded, beat with vigour and regularity about 60 or 70 per minute, and continued so to beat for an hour—affording ample leisure for making the following observations, which answer to the above propositions :—

*Series I.**

1. The first sound was perfectly loud and distinct; and it was louder on the body of the ventricles than over the semilunar valves.

2. The second sound was subdued, and heard with some difficulty: and it was more audible over the semilunar valves than at the other parts of the heart, being sometimes distinct at the mouths of the arteries when inaudible on the body of the ventricles.

3. Pressure on the arterial orifices with the fingers or the stethoscope invariably stopped the second sound; and even slight pressure caused a whizzing or bellows murmur with the first sound.

4. The first sound was diminished, but not wholly suppressed, by pressing upon the ventricles with the end of the stethoscope (so as to curb or restrict their full contractile tension).

5. At each systole the sudden tension of the ventricles was such as to produce an *abrupt shock* to the finger placed on any part of them, with which shock the first sound exactly coincided.

6. The first sound was diminished, but not suspended, by thrusting the ends of the fingers into the auriculo-ventricular orifices; the ventricles contracting less, and irregularly (from the impeded influx of blood).

7. An incision being made into the left auricle, and the scalpel being passed into the ventricle, so as partially to destroy the mitral valve, and the blood being allowed freely to escape, the first sound continued to be heard with each contraction of the ventricle. See 9 *a*.

8. The sound continued, though the right auricle was completely cut open.

9. And, finally, though the finger was introduced into the left ventricle, and was made by pressure to prevent the influx of blood into the right;

*a*. Its character, however, was not so clear and smart as when the ventricles contracted on their blood;

*b*. Thirty or more contractions, the majority very vigorous, took place after the incision had been made.

*Series II**.

10. Before the pericardium was opened, *both* sounds were very distinctly heard.

* Present Drs. Williams and Arnott, and Messrs. Babington, Smyth, H. J. Johnson, Peregrine, Good.

† Present Drs. Williams and Macleod, and Messrs. Keate, Partridge, Malton, Goode, Seagrim, and others. The heart acted vigorously for an hour.

11. Both were also distinctly heard through the lung interposed between the heart and the end of the stethoscope.*

12. About two or three inches up the aorta from its origin, the second sound was heard, (but not the first,) alternating with the impulse as felt on the ventricles.

13. The second sound was decidedly more distinct over the origins of the aorta and pulmonary artery than on the body of the ventricles; and, in that situation, it was louder than the first sound at the same point. It had exactly its natural short, clear, flapping character.

14. The aorta and pulmonary artery being compressed between the fingers, the first sound was accompanied with a loud murmur, and the second was stopped.

15. A common dissecting hook being passed into the pulmonary artery, so as to prevent the closure of the semilunar valves, the second sound was impaired, and a hissing murmur accompanied it. A hook being passed into the aorta, so as to act in the same way on the aortic valves, the second sound entirely ceased, and was replaced by a prolonged hissing. (Heard by several.)

16. When the hooks were withdrawn, the second sound returned and the hissing ceased.

17. Experiment 15 was repeated, and whilst Dr. Hope listened, the hook was first withdrawn by Dr. Williams from the aorta. Dr. Hope immediately said, "I hear the second sound."

18. Dr. Williams then removed that from the pulmonary artery; Dr. Hope said, "the second sound is stronger, and the murmur has ceased." (Several listened to 16, 17, and 18.)

19. The arteries were cut open: the heart continuing to contract (about eight or ten times), the first sound only was obscurely audible.

*Conclusions.*

These experiments appear to warrant the following conclusions:

A. The first sound is loudest on the body of the ventricles (1).

B. The second sound is loudest over the sigmoid valves, and thence for a few inches along the aorta (2, 12, 13.)

C. Preventing the reaction of the blood on the sigmoid valves, annihilates the second sound (3, 14.)

D. Creating regurgitation through the sigmoid valves occasions a murmur and extinguishes the second sound (15, 16, 17, 18.)

From the two last propositions it results that the closure of the sigmoid valves is the cause of the second sound.

E. The jerk (impulse) felt on the ventricles is coincident with and occasioned by the closure of the auriculo-ventricular valves, by which a sudden resistance is offered to the ventricular contraction (5): under these circumstances the first sound is perfectly loud and distinct (1, 10.)

F. When the resistance of the valves is removed, and the jerk thus prevented, the first sound is dull and obscure, (7, 8, 9, 19,) like the muscular sound which may be imitated with the hand.

* I made this observation to refute the contrary opinion held by M. Majendie.

The two last propositions seem to warrant the inference that the first sound is compound: viz. consisting, 1st, *possibly* of a degree of valvular sound; 2d, of a loud, smart sound produced by the abstract act of sudden jerking extension of the muscular walls, in the same way that such sound is produced by similar extension of the leather of a pair of bellows;—to avoid circumlocutions, I shall call this the *sound of extension:* 3d, a prolongation, and possibly an augmentation, of this sound by the sonorous vibrations peculiar to muscular fibre (*bruit musculaire, muscular sound.*)

These heads will severally require a few remarks. The *valvular sound* can only be spoken of as *possible*, because being synchronous with, and as it were incorporated in, the sound of extension, its existence is not demonstrable. The *possibility* may perhaps amount to a *probability*, on the principle that, if the sigmoid valves can produce sound, the same may, by analogy, be predicated of the auriculo-ventricular valves.

The existence of the *sound of extension* appears to me to rest on strong grounds. The phenomena in No. 5 made a forcible impression on all present; and it was remarked that the sense of touch conveyed an identical idea with the sense of hearing. The first sound of the heart during palpitation is, in some instances, of such extraordinary intensity, that it would do violence to all analogy to suppose it produced solely by *bruit musculaire;* and some of the strongest advocates for this *bruit* have thought it necessary, in such cases, to imagine a sound produced extrinsically, by the heart impinging against the thoracic walls. This corroborates the opinion which I formerly expressed, (page 47,) founded upon, and subsequently confirmed by, every variety of experiment on muscular sound which I could devise. Nor can it be supposed, again, that the auriculo-ventricular valves alone could produce so loud a first sound as is sometimes heard; not to say that this sound is of a different character, being much more blunt than the loaded valvular click, taking that of the sigmoids as a type.

The *bruit musculaire* appears to constitute the prolongation of the first sound, this prolongation being of the same dull, rumbling character as ordinary muscular sound. Possibly it may add to the intensity of the first sound. Nor can it be affirmed that the motion of the blood does not also contribute to the sound, though there is no proof that it does. In the healthy human subject, when faint, the first sound is short and flapping, like the second. For this, *bruit musculaire* alone would not account, as languor would increase the characteristic dulness and obscurity of the *bruit musculaire.* The first sound during faintness, therefore, must proceed from muscular extension, or from the valves, or from both.

Until the view now offered has passed the ordeal of professional scrutiny, it would be premature in me to venture upon alterations of the text to correspond with it: more especially as a most flattering reception in this country, and several foreign editions and translations, have given the present work a sanction which I am bound to respect. Now, as before, "I am anxious to offer my opinions, not as established facts, though I trust that they will be found grounded on careful observation, but simply as propositions to be admitted or rejected according to the test of general experience*." In the interim, the following brief remarks will afford a key to all the points in this volume, where the reader is to exercise his own judgment in applying the present view, or that previously offered, in explanations of the signs of disease. Happily, as Nature is immutable, the signs themselves are always the same, howsoever we explain them; but a correct explanation renders them more distinct and available.

*Sounds in Hypertrophy* (page 50).—As the ventricle, from its thickness, contracts slowly, the closure of the auricular valves is slower, is attended with a less jerk of extension of the ventricular walls, and the muscular sound (*bruit musculaire*) is weaker. Hence, the first sound is dull and stifled. The second sound is weaker because the diastole, no less than the systole, being performed more slowly, the reaction of the blood on the sigmoid valves is less smart.

*Sounds in dilatation* (page 52).—As the muscle, from being thin, contracts with increased velocity, the sound of muscular extension and of valvular closure is brief, loud, and clear; and it is not prolonged by *bruit musculaire*, or motion of the blood, apparently in consequence of the feebleness of the contraction. The second sound is increased from the diastole being quicker.

*Hypertrophy with dilatation* (page 53).—The first sound is increased, sometimes exceedingly, in consequence of the violence and velocity of the muscular extension and valvular closure: the sound is also prolonged by *bruit musculaire*, &c. The second sound is increased from the diastole being quick, but still more perhaps from the preternatural quantity of blood injected into the arteries increasing their tension, and consequently their reaction on the sigmoid valves.

*Disease of the valves* (page 55).—Murmur with, or instead of the second sound over the sigmoid valves, indicates regurgitation, either from disease of the valves or from their being too small to close the orifice.

### ON THE REDUCTION OF STRANGULATED HERNIA.

A memoir upon this subject by Dr. Herpin, read before the Medical Society of the Canton of Geneva, January 11, 1832, has been printed in the Gazette Médicale of May 16, 1835. The author remarks, that until the beginning of the nineteenth century the progress of surgery consisted chiefly of the improvement of operations. Of late years, however, much has been done to save patients from the knife, and many white swellings, diseases of the

* Introduction, p. 12.

breast, affections of the testis, &c. which would have been operated upon twenty years ago, are now cured by active medical treatment.

Among the diseases which appear exclusively to belong to the domain of surgery, but to which medical treatment is applicable, we must reckon strangulated hernia; and it is the object of this essay to show that the operation for its relief is too often practised. The treatment recommended by the old masters of the art, before recourse is to be had to cutting instruments, is very insignificant; and even in our own days the most eminent surgeons, though they recommend more therapeutical means, recommend them without confidence, and practically trust only to the operation. Dr. Herpin, after quoting Boyer's opinions on this point, (*Traité des Maladies Chirurgicales, tom.* ix. *p.* 90, 91.) remarks that if we wish to know Dupuytren's, independently of his clinical lectures, we may find them in the additions to the *Médecine operatoire* of Sabatier in the edition published under his auspices by his pupils MM. Bégin and Sanson. The authors after enumerating the different remedies advised against strangulated hernia, but which they consider applicable rather to incarceration than to inflammatory strangulation, namely, purgatives in repeated doses, tobacco clysters, resolvent or astringent topical remedies, and ice, proceed as follows: "General bleedings, the repeated application of leeches to the part, baths, emollient poultices, and abstinence from food and drink, bring the parts into a condition more favourable to reduction. This operation may then be attempted with much less inconvenience to the parts, and much greater chance of success. Nevertheless, as these means are far from infallible, and time is precious, antiphlogistics must be employed in general with a sort of mistrust, and the taxis with much reserve, and if the symptoms either are or become at all alarming, we must have recourse to those means which directly destroy their cause, and perform the operation."

Thus we see that the treatment which is to prevent the necessity of the operation is always represented as but little likely to be successful, and we are instructed to operate as soon as possible. The practice at the Hôtel Dieu, when our author attended there in 1820, was entirely in accordance with these precepts; reductions of hernia by the taxis were very rare; the pupils sometimes made the attempt before the arrival of the Professor, but he operated immediately. It is true that the majority of the patients did not arrive at the Hôtel Dieu immediately after the appearance of the symptoms, and must have undergone some attempts at reduction before; but had these always been sufficient, methodic, and preceded by proper treatment? It may be permitted to doubt this, and the want of success ought not to have prevented another trial. However this may be, every hernia, with very few exceptions, was operated upon without continued attempts at reduction. In many instances it was not even tried. I might quote many cases, says Dr. Herpin, in proof of these assertions, but they would be almost always a repetition of the same thing. I shall content myself with one instance, copied word for word from my notes.

"Melon (Germain), aged 18, was admitted into the Hôtel-Dieu on the 26th of February, 1820, and was placed in St. Bernard's ward, No. 57. When seen in the evening, he was found to have a hernia, which had been strangulated for twenty-four hours. Unsuccessful attempts at reduction had been made out of the hospital; the tumour was hard, painful, and formed a knuckle (*bourrelet*) beyond the ring. Without attempting reduction, M. Dupuytren immediately decided upon operating. After the incision of the integuments and other superficial structures, the operator," &c.

The remainder, says Dr. Herpin, does not concern our present purpose. The operation succeeded, and in six weeks the patient went out cured, having experienced but trifling symptoms during his recovery.

Imbued with the lectures which he had heard, and the examples that he had seen, our author, when he began practice, thought that he should be obliged to operate in the majority of the cases of hernia that came under his care. The fact, however, has been quite different.

From 1824 to January, 1832, Dr. Herpin treated seventeen cases of strangulated hernia. These seventeen cases occurred in only eleven individuals; for one patient had three, and another five. These were women who worked very hard, and said that they could not bear the strong trusses ordered them. Of the eleven patients, six were females, and five males; their ages were as follows:

| | | |
|---|---|---|
| 1 | was | 19, |
| 2 | were from | 30 to 40, |
| 1 | from | 40 to 50, |
| 5 | from | 50 to 60, |
| 2 | from | 60 to 70. |
| Total, 11. | | |

Among the women, all the herniæ were crural; among the men, all were inguinal, with one exception.

Of the seventeen herniæ, sixteen were really strangulated; one alone was merely incarcerated.

*Treatment.* In three cases the taxis alone, employed immediately, and with some perseverance, succeeded. In the case of the hernia incarcerated, the taxis was preceded by resolvent applications. In twelve cases reduction was effected by means of the bath, employed after the application of leeches, which was sometimes preceded by bleeding. In one case alone, (which was the fourth that occurred in Dr. Herpin's practice,) the operation was necessary. The patient was a peasant, aged about forty, who had fallen ill at an inn; the strangulation had lasted sixty hours: it was a crural hernia, small, and very painful. A bleeding, a bath, and leeches, having been employed to facilitate the taxis, but without success, our author operated, with the assistance of M. Senno, seventy-two hours after the occurrence of strangulation. The recovery was very rapid.

Subtracting, then, the incarcerated hernia, and the three cases where the taxis succeeded immediately, there remains but one operation, performed among thirteen herniæ, that were indubitably strangulated: yet, at the time of reduction, the strangulation had lasted various periods, up to forty-eight hours. Here is a case at length.

—— Tranchant, a married woman, aged about sixty-five, of a lymphatic temperament, small stature, decrepid and lame, and living in extreme indigence, had had a crural hernia for about ten years: it had been produced by a kick upon the abdomen. She did not wear a truss, but the hernia always went up with facility.

On the 5th of October, 1824, at eight in the evening, she carried a heavy saucepan to the fire, and, on going to bed two hours afterwards, she found that the hernia had come down, and tried in vain to reduce it: her husband also made useless attempts, with the same purpose. She soon had pains in the abdomen at intervals, and vomited. The pains continued on the 6th and 7th, and she vomited whenever she drank.

Our author was called in on the 7th, at one P.M., and found a crural hernia, as big as a pigeon's egg; its transverse diameter being the greatest. The tumour was smooth, hard, and not very painful on pressure; there were abdominal pains and vomiting, as on the preceding day, with thirst, a dry tongue, hot and dry skin, and a small, frequent, and rather hard pulse. Our author having endeavoured, but in vain, to reduce the hernia, ordered twenty leeches to be applied to the tumour, and infusion of mallow to be taken for drink.

At ten in the evening, the leech-bites had bled freely, and were still running a little. The abdominal pains had been much augmented by Dr. Herpin's attempts; the tumour was harder, and there were vomitings. The patient was put into a bath which had been got ready for her, and, having remained in it for forty minutes, felt very much indisposed, when the author renewed the taxis: at the end of a few minutes he heard a gurgling, and almost immediately afterwards the hernia was reduced. The strangulation had lasted more than forty-eight hours.

On the 8th, the patient had passed a liquid stool very soon after the reduction, and the pains had completely ceased: the hernia had come down during the night, in spite of the compresses and the spica bandage round the groin, (which Dr. Herpin had applied temporarily,) but it returned immediately. There was no vomiting; the thirst was less, the tongue moist; no appetite. Rice-water and low diet were prescribed. On the 13th, the patient had recovered perfectly.

This was the second case of strangulated hernia occurring in

Dr. Herpin's practice, and his success in treating it made him from that time persevere, much more than authors recommend, in attempts at reduction. He has always had reason to congratulate himself upon it, though he has sometimes met with such difficulties that he has no hesitation in believing that the majority of the patients, whom he has cured without operation, would have undergone one in the hands of practitioners much more skilful than himself.

The preceding case shows the treatment which he employs, which, however, he gives more at length, as follows; observing that it contains nothing new, excepting perhaps the combination of the remedies, and the time which is chosen for reduction.

Immediately after his arrival, the patient being properly placed, with the pelvis higher than the remainder of the trunk, the thighs bent, &c., Dr. Herpin tries the taxis. If the resistance is too great, he bleeds or not, according to the constitution of the indi dual; but he always orders leeches to be applied to the tumour, and a bath. The bath is generally ready the moment that the leeches fall off, in which case the patient is immediately to be put into it: if not, a poultice is applied to the leechbites during the interval. While the patient is in the bath, the clots are to be occasionally removed from the bites, and the blood generally flows with ease. The patient is left in the bath until he feels the uneasiness which precedes fainting: this almost always happens before he has been in the bath an hour. The taxis is now tried again: sometimes it succeeds immediately, sometimes the attempts are continued for half an hour, or even more, allowing the patient to repose from time to time. Dr. Herpin occasionally uses a good deal of force, and is not stopped by the painful state of the tumour; and, as already mentioned, this practice has been crowned with success twelve times out of sixteen. The patient does not faint, because the pain is sufficiently stimulating, but still there is a state of weakness which remarkably facilitates the reduction.

Thus, instead of bleeding to syncope, as Boyer advises, which it is not always easy to do, and which moreover would cause a much more lasting debility, the author employs the bath and evacuation of blood, with the same end in view; and, then, instead of taking the patient out of the bath, as Boyer recommends, he tries the taxis while the patient is in the bath. This is perhaps rather more troublesome to the surgeon, but infinitely better for the patient; for, while he is getting out of the bath, and being dried and shifted, the favourable moment is lost.

The author again observes that he uses a good deal of force when attempting the taxis in the bath; and quotes a case which has contributed not a little to make him persevere in his attempts at reduction. He was called into the country, some years ago, by one of his colleagues, to see a middle-aged farmer labouring under strangulated hernia. The operation seemed the only resource, and they took the necessary instruments with them. The strangulation had lasted about thirty-six hours. Bleedings, leeches, baths, and poultices had been employed in vain, from the preceding day to the evening; the tumour occupied the scrotum, and was large, hard, and painful. We both tried the taxis, says the author, without success (the patient being in bed) and then retired to consult together, having hinted to the patient that an operation would probably be necessary. The result of our conference was that it was requisite to operate instantly. Just as we were going in to tell the patient what we had resolved upon, he cried out that the rupture had gone up, and so it really had. *Crever pour crever*, said he, I squeezed it between my hands as hard as I could, (he was a strong man,) and it went up. The patient got well without a bad symptom.

The woman whose case was given above, was the patient whose recovery was the slowest, though she was perfectly convalescent five days afterwards; but she was indigent, and of a very bad habit of body, the strangulation had lasted more than forty-eight hours, and the subsequent diarrhœa alone kept her two or three days in bed. In the greater number of cases Dr. Herpin was not able to keep the patients from resuming their ordinary occupations on the following day.

What are we to think now, asks the author, of the advice which is given us to discontinue the taxis when the tumour becomes painful, to employ antiphlogistics with a kind of mistrust, and the taxis with much reserve? Nay, we are told that an immediate operation is generally to be preferred to prolonged attempts at reduction. I have allowed facts to speak for themselves, and leave others to decide if the authors referred to do not go too far,

We are not to conclude, however, that the operation may be put off to an indefinite period; the following is Dr. Herpin's summary of his opinions on this point.

1. That neither previous attempts at reduction, nor twenty-four, thirty-six, or forty-eight hours of strangulation, nor even the painful state of the tumour, contra-indicate the persevering use of the taxis, provided the patient is placed in the circumstances already pointed out; for even if it were necessary to operate immediately afterwards, these circumstances are a good preparation for the operation.

2. That independently of the pain of the operation, and five or six weeks of treatment, if we follow the ordinary theory and practice, we are sure to sacrifice a certain number of patients who are carried off by peritonitis.

3. That the treatment above described is the most calculated to obtain reduction,

There is an additional precaution which Dr. Herpin has sometimes found very useful in the taxis. When the tumour is tense, and very hard, he squeezes it for some time between the fingers of both hands, before endeavouring to return it; this compression must be gentle at first and gradually augmented. The tumour is almost always immediately softened, either from the pressure unloading the tissues, or from the return of a small quantity of gas.

### HISTORY OF A UNIQUE CASE OF HEART DISEASE.

By Samuel Hanna, A.M., M.B., one of the Physicians of the Sick Poor Institution.

Although I am fully aware of the general inutility of recording individual cases, except where by serving as types or models of a class they tend to direct our practice, yet I am induced to offer the following case, (which I met with several years since,) by reason of its singularity; as in the course of my subsequent experience I have happened on nothing approaching to it, nor on consulting a variety of authors, who treat expressly on diseases of the heart, have I been able to find any lesion similar, much less identical.

J. B., æt. 31, possessed of extraordinary bodily strength and activity, and of a restless, enterprising character; had been in the habit of living freely. He had met with several severe falls from horseback, having been formerly riding master to a dragoon regiment, and laterly being engaged in training horses. He, however, always enjoyed good health till the present attack; its origin he states, as follows:—On 25th of last August, he received a severe fall on his back from a horse, but found no immediate bad effects from it; however, in a couple of days he experienced a sudden beating at the heart, along with sickness; for this he took an emetic, (which aggravated the symptoms,) and from this period he has been subject to continual palpitations. A short time after, he heard something crack loudly to the left of the middle of the sternum, and there immediately succeeded in the same spot a burning pain, shooting occasionally under the scapula and down along the arm; this pain continued for a considerable time, but has since ceased during the use of digitalis. Sometime afterward, whilst hunting, he was suddenly seized with palpitations so severe as to induce fainting; this was relieved by a bleeding from the arm. He has spat blood. Such is the history of his case, as he detailed it to me. When I saw him he was much emaciated and enfeebled; the usual symptoms of heart complaint were present; dyspnœa on motion, especially up ascents, orthopnœa, palpitations, dreams, and startings, cough, &c. Pulse rather small, but regular. The signs furnished by the stethoscope were—Between the second and third ribs, near the sternum, is heard a loud whirring rush of a fluid along with a double beat; this is audible through the whole region of the heart, but is much loudest at the above point; and the finger applied here perceives a very marked thrill, ("frémissement cataire"). The force of impulsion at this point is various, sometimes much exceeding what is natural; and this not depending on palpitations. Percussion here returns a clear sound. I bled him, and put him on digitalis, hyoscyamus, &c. This occurred in the month of January. During the following month of February, the disease continued to make progress; the œdema of ankles increased, though it never was excessive; and the anguish from the feeling of suffocation became intolerable.

On the 1st of March, following the advice of a friend, he took some opium, (though from having witnessed, in two or three cases of heart disease, death notably hastened by its use, I had warned him against it;) when I visited him that evening, he said he was quite easy and happy; but he was then evidently under the delusive influence of the opiate. On calling the following day, I learned that shortly after my leaving him the preceding evening, he was seized with a fit of suffocation, and after ten minutes severe agony, had expired.—The following day I proceeded to make the examination of the body, but as several of the friends were present, the heart was the only organ I could inspect, and this too, hurriedly, as they were anxious to dispatch the funeral. The pericardium contained about a naggin, or a little more, of clear serum; the portion of it lining the heart was in parts dotted with red, and had here and there on its surface shreds of false membrane. The heart itself was two to three times its proper size. On making an incision from the origin of the aorta along the left ventricle toward the apex, I opened into a cavity, which I at first conceived to be that of the ventricle, but soon finding my mistake, and expressing my surprise at the appearance, I was permitted by the friends to remove the heart for further inspection. On returning home, I proceeded to examine the cavity: it might contain a small orange, and was formed in the external paries of the left ventricle; it was separated from the cavity of the ventricles by what seemed the inner coat, transformed into a thick fibrous membrane, while in the outer wall, the muscular texture of the ventricle was quite effaced, as if by the effect of compression; it was lined with shreds of coagulable lymph of various thickness, which easily peeled off. Just at the summit is seen a small, round, smooth opening, about two lines in diameter, leading at once into one of the sinuses of the aortic valve, and situated about four lines below the mouth of the coronary artery.

All the valves of the heart and of its vessels were sound; the aorta was of natural dimensions, and otherwise healthy, except in a diffuse partial redness in a small space of its inner coat, which, however, disappeared after twenty-four hours' maceration. On the surface of the interventricular paries of the fifth ventricle, is an exactly circular patch of a white colour, about the size of a shilling; this is formed by a softish layer of a plastic lymph, and is united only at its circumference to the lining membrane of the ventricle: corresponding to this patch the lining membrane has a shallow depression, and is more vascular than natural, shewing evident marks of inflammation.

On reading the preceding case, the question naturally arises as to the nature of the affection. It appears to me, to be an aneurism of the aorta developed in a most unusual situation; indeed on the whole, a lesion to which, after consulting a variety of authors, I

can find no precise parallel. The only question can be between this view of the matter, and an abscess in the substance of the left ventricle. Of this latter affection, there are not wanting several instances, but so imperfectly described, as to give little help in elucidating the question. Morgagni quotes three or four examples from the Sepulcretum of Bonetus; but on reference to that work, these will be found to be little more than mere statements of the existence of abscesses, without any anatomical detail. His object in quoting these cases, was only to ascertain, whether syncopes and intermissions of pulse were necessary symptoms of this affection, (a question by the way which he decides in the negative). Now, not to mention that abscesses, situated in the heart, are rather infiltrations of pus among the muscular fibres, it would be hard to conceive such a cavity left completely empty of pus, except we are to regard it as the consequence of the softening down and subsequent removal of a large tubercle, for these have not unfrequently been found in the heart's substance. But every thing in the history of the case, as well as in the constitution of the individual, disproves such an opinion; as he was of an eminently robust habit, and the attack was sudden, bearing in it all the characters of an acute inflammation. I may here also refer to the authority of Andral, who states, that he never found tubercles in the heart, except they existed at the same time in other parts of the body. In the case under consideration, there was no symptom of such, and the lungs, superficially examined, shewed no sign of their presence.

The heart is deposited in the museum of the Park-street School.

### ON THE TRICUSPID VALVE, OR SAFETY-VALVE.

A paper was lately read before the Royal Society, "On the Reflux of the Blood through the Right Auriculo-ventricular Aperture of the Human Heart," by T. W. King, Esq.; which was communicated by Thomas Bell, Esq. F.R.S. The author of this paper shows, as the result of the anatomical arrangement of the valves of the heart, that, whilst the mitral valve is true and powerful in its valvular function, the tricuspid valve performs the office both of a valve and of a safety-valve, as occasion requires: that is, it is either nicely closed against all reflux, or it is widely open, expressly to admit a free regurgitation, under different circumstances.

Pathologists have long known that dilatation of the ventricle so deranges the attachments of the mitral and tricuspid curtains as to produce an imperfect valvular action; and it has been more or less allowed that the action of the right side of the heart is less perfect and forcible than that of the left. It appears, however, that the loose thin wall of the right ventricle has so much of the tricuspid curtain attached to it, that all degrees of congestion, even in health, tend to diminish the nice adjustment of the naturally feeble valve. In the common congestion of death, the valve, when set in action by experiment, presented an aperture of reflux nearly equal in area to the tube of the pulmonary artery. It appears, also, that the tricuspid is only a true valve when the ventricle is nearly in a maximum state of contraction.

Independently of the anatomical explanation of this new function, the proof rests upon a simple experiment which has been performed in various ways upon living and dead hearts: it consists in this: The heart being removed from the body, and the great arteries divided a short distance from their origin, the sigmoid or semilunar valves of both are to be destroyed, and injection-pipes are to be inserted, so that the ventricles may be injected. The auricles are next to be fully removed, in order to expose the valves; and now, upon injecting the ventricles in succession, from the aorta and pulmonary artery, it will be found that the mitral curtains always close perfectly and powerfully; but, on the contrary, the tricuspid do not close in any degree accurately under common circumstances.

It seems that the more the right ventricle has been distended in death, the greater is the reflux, and *vice versâ;* that, in living animals of various kinds, the least fulness of the cavity produced by the injection of warm water, gives a copious refluent stream, and that the living hearts of men, and many mammalia, if allowed fairly to contract after death, are then found to possess a true valvular action in the right ventricle. Thus, it is shown that, in proportion as the right ventricle is distended, whether by impediment towards the lungs or general venous fulness, this safety-valve operation prevents the undue propulsion of blood to the lungs, whilst the auricle and veins act as reservoirs, until the left side of the heart and lungs can gradually dispose of the increased mass of fluid, when, finally, the right ventricle is unable to adapt its dimensions to the reduced contents, and the more perfect valvular office comes into operation.

The author states that, in fœtal life, the solidity of the right ventricle both maintains and needs a more perfect valve; but that, after birth, when the nutrition and movements at length become irregular, and sometimes violent, the safety-valve action becomes superadded upon the wasted ventricle. He advances the probability that males have a fuller safety-valve action than females; and he also shows that it is likely, from the various forms of the tricuspid valve in different animals, that the several orders of the class mammalia have each more or less safety-valve action.

The anatomical descriptions of these parts were facilitated by drawings; and the characters of the heart at different ages, and of the organ in different animals, as well as the circumstances of disease which involve the safety-valve function, were distinctly noticed.

ST. GEORGE'S HOSPITAL.

*Fractured Pelvis ; Hemorrhage ; Suppuration within the Pelvis.*

Charles White, aged seventeen, was admitted, January 13th, under the care of Mr. Cæsar Hawkins, having just before been knocked down upon his face by a horse; a loaded dung-cart having then passed over the back of the pelvis. On his first admission, he appeared to be dying of internal hemorrhage, with much pain and distention of the abdomen: it was thought, however, that the bleeding was probably external to the peritoneum within the pelvis, as the pain and swelling were chiefly at the lower part of the abdomen. No blood, however, appeared externally, except on the back of the nates, which were much contused. In a few hours, however, he recovered from the hemorrhagic collapse, though still complaining of much pain. He had made water an hour before the accident, and was unable to make water himself, on account of a rupture in the urethra. A gum-elastic catheter was in the evening passed with some difficulty into the bladder, through the lacerated part, in which there seemed to be a good deal of coagulum; and it was fastened in the bladder, the water in which was quite clear, and free from blood.

Jan. 14th. Seems comfortable, except from pain in the lower part of the abdomen, which was hard and tense, as if from blood in the muscles. Pulse frequent and sharp, as if from hemorrhage; bowels confined.—℞. Cal: gr. iij.; Jalapæ, gr. xvi. M. statim sumend.

15th. Tolerably comfortable: skin rather hot; less tendern ss and pain. -℞. Haust. Salin. ℥iss.; Magnes. Sulph. ʒss. M. teis horis sumend.

Examination of the pelvis gave the sensation of obscure crepitus, as if of cartilaginous surfaces rubbing against each other in the right iliac synchondrosis, and a sensation of crepitus was also perceptible in the front of the pubes near the lacerated part of the urethra. Motion, especially of the right leg, gave him also very great pain.

16th. He last night began to complain of more pain in the abdomen, and to-day has much tenderness and pain above the pubis: this continues to-day, and he has an expression of much anxiety of countenance, with a small thready pulse, at 116, and fever: bowels open. Mr. Hawkins believed that there was inflammation of the lower part of the peritoneum, and directed him to be bled, which was done to ten ounces. This gave him much relief, and the pulse became fuller and stronger, the blood being much buffed and cupped.

The water now containing some mucus from the bladder, Mr. Hawkins withdrew the catheter, and found it incrusted with phosphates. A new one was then passed in, and the bladder was injected with warm water once a day; from which he experienced much benefit, and the urine became again clear.

Vespere. Pain continues, though less.—Hirud. abdomini.—

℞. Hydr. Submur. gr. ij.; Opii, gr. $\frac{1}{6}$th. M. fiat pil. quartis horis sumenda.

17th. Seems a good deal better, but the pulse has still some sharpness, and the pain is not gone.—V.S. ad ℥viij. Blood cupped and buffed, though less. Cont. pil.

18th. All expression and anxiety gone; much less pain and tenderness. Catheter again changed, and found slightly incrusted with phosphate of lime; a little mucus having also again been secreted in the urine.—Cont. pil. 8vis horis.

19th. Omit. pil. Beef-tea, half a pint.

21st. Still going on well, and free from pain.—Fish diet.

22d. Does not seem so well, being weak and nervous, and losing his appetite, though not complaining when not moved. A good deal of fœtid bloody pus coming away by the side of the catheter, and no difficulty being experienced in making water when this was tried, the catheter was withdrawn, and he was allowed to make water, so that the pus was not confined, and no irritation excited in the bladder, of which he made much complaint. Bowels somewhat purged, and the evacuations fœtid and dark. Took castor-oil yesterday.—℞. Calomel, gr. iij.; Opii, gr. ss. M. statim.—℞. Haust. Rhei, ℥j. post hor. iij.

23d. Same symptoms, but increased; restless and anxious; unable to take any food; complains of much pain in the abdomen and perineum, in which last situation no swelling could be perceived; much bloody purulent fluid coming away by the urethra and by the catheter, the point of which entered a cavity communicating with the perineum, though not appearing externally, so that Mr. Hawkins believed that suppuration had taken place on the inside of the pelvis where the blood had been extravasated, probably about the period when the peritonitis had come on. He therefore passed a catheter with some difficulty into the bladder, the water in which was still quite clear, and made a large incision in the perineum, the cavity in which was not large; but an opening was found on the right side, going into the interior of the pelvis, which was enlarged by the knife, and a large quantity of the same putrid bloody pus was let out. The finger, passed into the pelvis through the opening, detected an extensive fracture across the thyroid foramen, on the right side, the broken portions being partly loose, and extensively deprived of periosteum, in the cavity from which the fluid had been evacuated.

Wine, arrowroot, &c. were given, but the prostration of strength went on increasing; the next day he had much vomiting, and died in the evening of the 24th.

*Examination of the body.* Peritonitis only at the lower part of the abdomen, where the sigmoid flexture of the colon, the omentum, and part of the small intestine, were firmly adherent to the peritoneum lining the parietes, and covering the bladder; and the bowel at this part was very soft, and easily lacerated. A large quantity of blood was extravasated on the outside of the peritoneum, chiefly around the pelvis and back of the abdomen, the peritoneum being every where raised and separated from the adjacent parts, the blood having even made its way along the mesentery to the coats of the intestines, and extending in the same way into the rectum and colon. The bladder was contracted and small, though uninjured, in the midst of a mass of coagulum above an inch thick, between the peritoneal and muscular coats, and nearly as much in front. The blood had also been driven a little into the substance of the iliac and psoæ muscles on both sides, and someway up the anterior parietes of the abdomen, chiefly, however, between the peritoneum and transversalis. There was very little blood in the adductor muscles of either side, and very little in the perineum, so that, although the source of the bleeding could not be ascertained, it did not appear at all likely to have been effused from the ruptured urethra, but from some of the vessels of the interior of the pelvis injured by the fractured bones.

The laceration of the urethra had taken place just under the symphysis, and was probably occasioned by the fracture of the left side.

The front of the pelvis, between the bladder and innominata, and deep in the pelvis, was occupied by a large cavity in a sloughy state, which reached up also some little way on the front of the abdomen. The suppuration had not extended, however, to the blood on the posterior part of the bladder, or among the intestines. In this cavity much of the fractured bones on the right side lay nearly loose and dead, so that the adductor muscles formed the boundary of the abscess. The right os innominatum was broken into several portions through the thyroid foramen, the transverse ramus of the pubes being separated at the symphysis, and loose and dead from the line of fracture. The ascending branch of the ischium was broken separately, nearly down to the tuberosity. The left innominatum was broken across the thyroid foramen in the same way, though to a less extent, and none of it seemed deprived of periosteum by the sloughing abscess. The ilium was separated from the sacrum at the synchondrosis on both sides, where a little blood had also been effused, but evidently from small vessels only, quite distinct from the great extravasation. On the left side a small portion of the sacrum had been broken off at the joint.—*Med. Gazette.*

---

